# Efficacy evaluation and clinical value exploration of secondary inpatient treatment for total deafness-type SSNHL: a single-center prospective study

**DOI:** 10.3389/fneur.2026.1745064

**Published:** 2026-02-02

**Authors:** Yong Li, Ziyuan Chen, Yilong Wang, Yongjie Ying, Changyu Duan, Qiaozhi Jin

**Affiliations:** 1 Department of Otolaryngology, Taizhou Municipal Hospital (Taizhou University Affiliated Municipal Hospital), Taizhou, China; 2 School of Medicine, Taizhou University, Taizhou, China; 3 Department of Otorhinolaryngology-Head and Neck Surgery, The First Affiliated Hospital of Fujian Medical University, Fuzhou, China

**Keywords:** hearing outcome, nomogram, prognostic predictor, secondary inpatient treatment, total deafness-type SSNHL

## Abstract

**Background:**

Total deafness-type sudden sensorineural hearing loss (SSNHL) represents one of the most challenging subtypes of SSNHL due to its poor response to initial therapy and uncertain prognosis. Secondary inpatient treatment has been proposed as a potential salvage strategy; however, its efficacy and predictors of favorable outcomes remain poorly defined.

**Methods:**

This study included 120 patients with unilateral total deafness-type SSNHL, divided into secondary treatment and control groups. Hearing thresholds at low, middle, high, and full frequencies, pure-tone average (PTA) at speech frequencies, and speech recognition rate were evaluated across six time points (T1–T6). Tinnitus Handicap Inventory (THI) scores and improvement rates were also analyzed. Univariate and multivariate logistic regression analyses were performed to identify independent predictors of marked hearing recovery. A nomogram was constructed to predict the hearing prognosis of patients with SSNHL.

**Results:**

Compared with the control group, the secondary treatment group exhibited significantly earlier onset and greater magnitude of improvements in hearing thresholds and speech recognition rate (all *p* < 0.05), with distinct frequency-specific patterns. Recovery initiated at 4–8 weeks and stabilized after 12 weeks, while the control group showed delayed improvement. Tinnitus relief occurred earlier in the secondary treatment group. Multivariate analysis identified age ≤50 years, disease duration ≤3 days, absence of vertigo, and normal vestibular function (vHIT and caloric test) as independent predictors of marked recovery (all *p* < 0.05). The area under the receiver operating characteristic (ROC) curve was 0.876 (95% confidence interval [CI]: 0.762–0.989). The calibration curve showed good agreement with the standard curve. The decision curve analysis demonstrated that the prediction model yielded positive net benefits across nearly all threshold probability ranges.

**Conclusion:**

Secondary inpatient treatment offers a significant auditory benefit for patients with total deafness-type SSNHL by accelerating and amplifying recovery. Young age, early intervention, and well-preserved vestibular function are key determinants of a favorable prognosis. The predictive model constructed hereby can effectively predict the prognosis of patients.

## 1 Introduction

Sudden sensorineural hearing loss (SSNHL) constitutes a clinically significant otological emergency, defined by rapidly developing idiopathic sensorineural hearing impairment within 72 h, presenting as a hearing threshold decline ≥30 dB HL affecting at least 3 contiguous frequencie (1). Epidemiological trends demonstrate a progressive increase in SSNHL incidence, correlating with modern lifestyle acceleration, elevated psychological stress levels, and worsening acoustic environmental pollution. Current global prevalence estimates range from 5 to 20 cases per 100,000 population, with a noticeable demographic expansion from primarily middle-aged and elderly individuals to younger adult populations (2). As hearing represents a fundamental sensory function, its sudden impairment not only disrupts verbal communication abilities but also predisposes individuals to psychological comorbidities such as anxiety and depression, potentially leading to social withdrawal (3–5). The marked deterioration in quality of life substantiates SSNHL’s evolving status as a pressing public health concern demanding immediate attention (6, 7). However, therapeutic management of the profound subtype remains particularly challenging.

SSNHL manifests through four distinct audiometric configurations determined by frequency involvement and severity parameters: low-frequency descending, high-frequency descending, flat, and profound hearing loss subtypes, each exhibiting characteristic pathophysiological mechanisms and prognostic pathway (8). The total deafness-type represents the most severe clinical variant, (9, 10) characterized by average hearing thresholds ≥81 dB HL across the 250–8,000 Hz frequency range and generally poor auditory recovery outcomes (8).

Despite current clinical guidelines recommending combination therapy comprising systemic glucocorticoids, microcirculation-improving agents, and neurotrophic factors as primary intervention for profound SSNHL, therapeutic efficacy remains considerably lower compared to other subtypes (1, 2, 8). Multicenter investigations report insufficient initial treatment response (hearing improvement <15 dB HL) in over 70% of profound SSNHL patients (10, 11). These treatment-resistant cases encounter multifaceted challenges including permanent hearing disability, communication deficits, and frequent co-occurrence of persistent tinnitus and vestibular dysfunction. The resulting psychological distress and diminished quality of life generate substantial burdens for healthcare infrastructure and societal resources (12, 13). Consequently, formulating effective salvage therapeutic approaches for non-responding patients and identifying reliable prognostic indicators represent urgent research priorities in modern otology (14).

Considerable debate continues regarding the clinical utility of secondary interventions for SSNHL, with insufficient high-quality evidence guiding treatment decisions. Initial research efforts have examined the feasibility and refinement of salvage treatment regimens, yet methodological constraints prevent conclusive clinical recommendations. Prevailing study methodologies employing single-timepoint audiometric evaluations fail to document the dynamic progression of hearing recovery, thus yielding incomplete assessments of therapeutic effectiveness (15–17). The lack of established predictive biomarkers for secondary treatment outcomes additionally hinders appropriate patient selection, potentially excluding suitable candidates from beneficial interventions while exposing others to unnecessary treatment risks and healthcare costs.

Contemporary research primarily emphasizes short-term treatment outcomes, paying inadequate attention to delayed therapeutic responses and long-term stability (18, 19). Moreover, the persistence of hearing recovery remains insufficiently examined, particularly concerning potential late-phase hearing regression (20, 21). This knowledge deficiency obstructs the formulation of evidence-based long-term management approaches and thorough efficacy appraisal.

This research initiative incorporated 120 eligible patients with unilateral profound SSNHL, randomly allocated to secondary treatment or control groups. Seven predetermined evaluation timepoints spanning from baseline to 24 weeks post-treatment enabled thorough assessment using combined audiometric measurements, speech recognition scores, tinnitus handicap evaluation (THI), and vestibular function tests (vHIT and caloric testing). Our investigation introduces novel temporal analysis of frequency-specific hearing recovery dynamics, permitting scientifically informed efficacy judgments and structured monitoring schedules to avoid inappropriate therapy discontinuation or excessive treatment. The enhanced assessment framework integrates speech perception and tinnitus metrics, enabling comprehensive appraisal of communication ability and life quality enhancements. We implemented a precision-based patient selection protocol recognizing age, symptom duration, and vestibular function as independent prognostic factors, followed by creation of a streamlined predictive algorithm to enable focused treatment allocation. Extended 24-week surveillance verified maintained therapeutic advantages, confirming the sustained effectiveness of secondary intervention and supplying compelling evidence for developing long-term management protocols. These outcomes collectively promote evidence-guided salvage treatment for profound SSNHL and anticipate improved clinical results.

## 2 Materials and methods

### 2.1 Study subjects

This was a single-center, prospective, non-randomized controlled study conducted in the Department of Otolaryngology, Taizhou University Affiliated Municipal Hospital, Taizhou, China, from January 2022 to December 2024. The study protocol was approved by the Ethics Committee of Taizhou University Affiliated Municipal Hospital (Ethics Approval No.: KTYJ2021000182) and was performed in strict accordance with the 2013 revision of the Declaration of Helsinki. Written informed consent was obtained from all participants before enrollment. Eligible patients who met the inclusion criteria and none of the exclusion criteria were recruited consecutively. Group allocation was based on the treatment strategy independently chosen by patients and their families after comprehensive informed consent about the potential benefits, risks, and logistics of each approach. Enrollment ended when 60 eligible patients had been recruited for each group, in accordance with the prespecified target sample size derived from an *a priori* power calculation. To minimize selection bias and ensure baseline comparability between groups, a dedicated research coordinator, who was independent of the treating physician team, was responsible for patient screening, verification of enrollment eligibility, and the systematic collection of baseline data, including demographic characteristics, disease-related variables, and audiological parameters.

The inclusion criteria in this study were as follows: (i) Meeting the diagnostic criteria for total-deafness-type sudden deafness outlined in the *Guidelines for the Diagnosis and Treatment of Sudden Deafness (2015 Edition)* issued by the Chinese Society of Otorhinolaryngology-Head and Neck Surgery, Chinese Medical Association, defined as idiopathic sensorineural hearing loss with onset within 72 h and an average hearing threshold ≥81 dB HL across 250–8,000 Hz (8); (ii) Unilateral involvement and age ≥18 years; (iii) Patients in the secondary treatment group were re-hospitalized for 1 week of secondary treatment 1 week after discharge from the initial hospitalization (i.e., 3 weeks after the first hospitalization date); (iv) Time from onset to initial hospitalization and treatment ≤7 days; (v) Completion of follow-up for 24 weeks after the first hospitalization, with complete audiometric and clinical data available at all predefined time points.

The exclusion criteria were as follows: (i) Achievement of “complete recovery” (hearing restored to normal, to the contralateral ear level, or to pre-onset level) at discharge after the initial 2-week treatment course; (ii) Presence of severe endocrine (e.g., hyperthyroidism, hypothyroidism), autoimmune (e.g., systemic lupus erythematosus), or hematological disorders (e.g., leukemia, aplastic anemia), or a history of diabetes; (iii) Significant functional impairment or organic disease affecting major organs such as the heart, brain, or kidneys; (iv) Malignant tumors, irrespective of prior radiotherapy or chemotherapy; (v) Coexisting cochlear nerve pathology (e.g., auditory neuropathy) or middle ear disorders (e.g., secretory otitis media, cholesteatoma); (vi) Prior diagnosis of Meniere’s disease, acoustic neuroma, vestibular neuritis, vestibular migraine, enlarged vestibular aqueduct syndrome, or intracranial space-occupying lesions (e.g., brain tumors); (vii) Cochlear implantation or other hearing rehabilitation interventions during the follow-up period; (viii) Incomplete clinical data (e.g., missing medical records, examination results, or follow-up data) or loss to follow-up.

### 2.2 Treatment regimens

#### 2.2.1 Initial inpatient treatment regimen

Both groups received standardized combination therapy during the initial 2-week hospitalization according to the *Guidelines for the Diagnosis and Treatment of Sudden Deafness (2015 Edition)*: (i) Systemic glucocorticoid therapy: Dexamethasone Injection was intravenously administered at 10 mg/day for 3 consecutive days, followed by 5 mg/day for an additional 3 consecutive days, after which the medication was discontinued; (ii) Microcirculation-improving therapy: Alprostadil injection 10 μg diluted in 10 mL normal saline, administered intravenously once daily for 14 consecutive days; (iii) Neurotrophic therapy: Methylcobalamin Injection (0.5 mg) diluted in 10 mL normal saline and administered intravenously once daily for 14 consecutive days; (iv) Batroxobin therapy: Initial dose of 10 Batroxobin Unit (BU) diluted in 100 mL normal saline, administered intravenously, followed by 5 BU every other day. Fibrinogen (FIB) levels were monitored prior to each administration; if FIB < 0.8 g/L, treatment was suspended for 1 day and resumed upon FIB ≥ 0.8 g/L. A total of 5–7 doses were administered during the initial hospitalization.

#### 2.2.2 Secondary hospitalization regimen

Patients were re-admitted 1 week after discharge from the initial treatment for a 1-week secondary course. The regimen was modified as follows: (i) Intravenous dexamethasone was replaced by post-auricular dexamethasone injection, with 0.7 mL Dexamethasone Injection mixed with 0.3 mL 2% Lidocaine for subcutaneous injection behind the ear every other day, totaling 3–4 injections based on patient tolerance, while other medications remained consistent with the initial regimen; no local adverse reactions such as pain, redness, swelling, or skin rash at the injection site were observed. (ii) The hyperbaric oxygen treatment protocol consisted of compressing the chamber to 0.2 MPa (2.0 ATA) over a 20-min period, followed by a 60-min interval of oxygen administration via a tight-sealing mask, and concluded with a 20-min decompression phase. This protocol was administered once daily. No adverse reactions were observed in patients upon completion of the treatment. (iii) Throughout the secondary treatment course, no systemic adverse events related to other medications (including Alprostadil, Methylcobalamin and Batroxobin) were reported.

#### 2.2.3 Maintenance regimen

After discharge, all patients, those in the control group and those in the secondary treatment group (both before and after secondary hospitalization), received oral maintenance therapy until 12 weeks after the initial hospitalization. The regimen included: *Ginkgo Biloba* Extract Tablets (80 mg, three times daily), Betahistine Mesilate Tablets (12 mg, three times daily), and Methylcobalamin Tablets (0.5 mg, three times daily). Patients with hypertension received standardized antihypertensive therapy under consultation with the Department of Cardiovascular Medicine to maintain blood pressure within normal limits (systolic <140 mmHg, diastolic <90 mmHg) throughout treatment.

### 2.3 Outcome measures and detection time points

#### 2.3.1 Pure-tone audiometry

Pure-tone audiometry was conducted in a soundproof booth compliant with GB/T 16403–1996 standards (background noise <30 dB(A)) using a Danish MADSEN Astera audiometer with TDH-39 headphones. Test frequencies included 250, 500, 1,000, 2000, 4,000, and 8,000 Hz. The maximum output intensities were 105 dB HL for 250 and 8,000 Hz, and 120 dB HL for 500–4,000 Hz. If no threshold was elicited at the maximum output, that maximum value was recorded as the threshold. Frequencies were categorized as low (250, 500 Hz), middle (1,000, 2000 Hz), and high (4,000, 8,000 Hz). The average hearing threshold for each frequency band is defined as the mean of the hearing thresholds at the two frequencies within that band, whereas the full-frequency (250–8,000 Hz) average hearing threshold is used for overall efficacy evaluation.

#### 2.3.2 Monosyllabic word recognition rate

The *Chinese Standard Speech Audiometry Word List* was used. Tests were conducted in a quiet environment (background noise <30 dB(A)). The pure-tone average of 500, 1,000, 2000, and 4,000 Hz (PTA-4) served as the reference for determining stimulus intensity. (i) For PTA-4 < 70 dB HL, the stimulus intensity was set to PTA-4 + 30 dB sensation level (SL); (ii) For PTA-4 ≥ 70 dB HL, stimulus intensity was fixed at 100 dB HL; (iii) If the interaural difference in PTA-4 was ≥40 dB HL, narrowband noise masking was applied to the contralateral ear at a level 20 dB lower than the stimulus intensity. Twenty-five monosyllabic words were presented per test, and the proportion of correctly recognized words (correct responses/25 × 100%) was recorded as the recognition rate, reflecting real-world speech perception ability.

#### 2.3.3 Tinnitus improvement

Tinnitus severity was evaluated using the Tinnitus Handicap Inventory (THI), consisting of 25 items across functional, emotional, and catastrophic dimensions. Each item was scored as “yes” (4 points), “occasionally” (2 points), or “no” (0 points), with total scores ranging from 0 to 100. Based on the total score, tinnitus handicap was classified as: Grade I (0–16, none), Grade II (18–36, mild), Grade III (38–56, moderate), or Grade IV (58–100, severe). A ≥ 20-point reduction in THI score from baseline was defined as “clinically significant improvement,” whereas a reduction <20 points or score increase indicated “no improvement.”

#### 2.3.4 Efficacy evaluation

Following the *Guidelines for the Diagnosis and Treatment of Sudden Deafness (2015 Edition)*, (8) treatment efficacy was categorized based on changes in the overall average hearing threshold relative to baseline: (i) *Complete recovery:* Hearing restored to normal (≤25 dB HL), the level of the contralateral ear, or pre-onset level;(ii) *Marked improvement:* ≥30 dB HL improvement; (iii) *Improvement:* 15–29 dB HL improvement; (iv) *No improvement:* <15 dB HL improvement. Total effective rate = (cases of improvement + marked improvement + complete recovery)/total × 100%; Marked effective rate = (cases of marked improvement + complete recovery)/total × 100%.

#### 2.3.5 Delayed hearing recovery

Delayed hearing recovery was defined as an improvement in hearing thresholds at any frequency at 24 weeks (T6) compared with post-initial treatment (T1), reflecting the delayed therapeutic effect.

#### 2.3.6 Detection time points

The first day of the initial hospitalization was designated as baseline (T0). Seven standardized detection time points were established to ensure consistency: T0 – baseline (before treatment); T1 – post-initial treatment (2 weeks after initial hospitalization); T2 – pre-secondary treatment (3 weeks, secondary group only); T3 – post-secondary treatment (4 weeks, or recheck time for controls); T4–8 weeks; T5–12 weeks; T6–24 weeks (final follow-up).

### 2.4 Comparative metrics

#### 2.4.1 Intragroup comparisons

Within-group comparisons were conducted for changes in low-, middle-, high-, and overall-frequency hearing thresholds, monosyllabic word recognition rate, and tinnitus between successive time points: T1 vs. T0, T2 vs. T1 (secondary group only), T3 vs. T1, T4 vs. T3, T5 vs. T4, and T6 vs. T5, to assess dynamic treatment responses over time.

#### 2.4.2 Intergroup comparisons

Between-group comparisons at each key time point included the following indicators: (i) Average hearing thresholds (low-, middle-, high-, and overall-frequency); (ii) Hearing threshold improvement relative to baseline (T0); (iii) Monosyllabic word recognition rate; (iv) Tinnitus improvement rate (proportion of cases with ≥20-point THI reduction); (v) Distribution of efficacy grades (no improvement, improvement, marked improvement, complete recovery).

#### 2.4.3 Subgroup comparisons

Within the secondary treatment group, patients were stratified by baseline clinical characteristics to identify subpopulations potentially benefiting most from secondary therapy. Comparisons of hearing threshold improvement at T6 were made among subgroups defined by: (i) Gender (male/female); (ii) Age (≤50/>50 years); (iii) Affected side (left/right); (iv) Time from onset to initial treatment (≤3 days/>3–≤7 days); (v) Presence of vertigo (yes/no); (vi) Caloric test results (normal/abnormal); (vii) Video head impulse test (vHIT) results (normal/abnormal). Caloric and Video head impulse test (vHIT) tests were conducted only for patients with vertigo to examine associations between vestibular function and hearing recovery.

### 2.5 Statistical analysis

Statistical analyses were performed using SPSS version 26.0. Measurement data were expressed as mean ± standard deviation (x̄ ± s). Within-group comparisons were performed using paired-sample *t*-tests, and between-group comparisons using independent-sample *t*-tests. Categorical data were presented as frequency and percentage (*n*, %) and analyzed using the χ^2^ test. Univariate analyses (*t*-test or χ^2^ test) were first conducted to identify potential predictors of hearing recovery. Variables with *p* < 0.05 in univariate analysis were included in a multivariate logistic regression model (dependent variable: delayed hearing recovery; 1 = yes, 0 = no) to identify independent influencing factors. A nomogram prediction model was constructed using the “rms” package in R software (Version 4.0). The discriminative ability and calibration of the model were evaluated using receiver operating characteristic (ROC) curves, calibration curves, and decision curve analysis (DCA). *p* < 0.05 was considered statistically significant.

## 3 Results

### 3.1 Comparison of baseline data between the two groups

**Figure 1:**
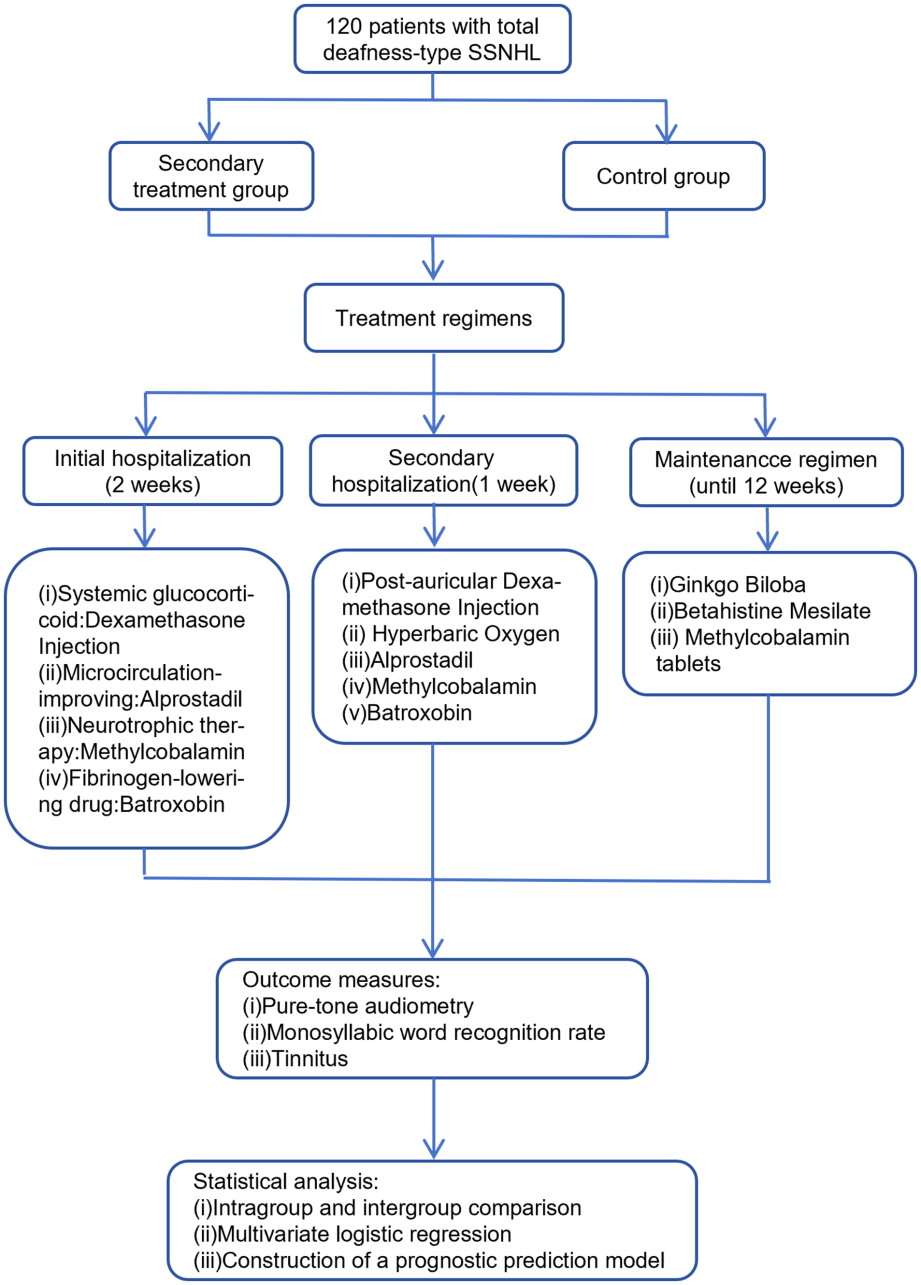
Study design and flowchart of secondary treatment for total deafness-type SSNHL.

A total of 120 patients were enrolled in this study, with 60 patients in each of the secondary treatment and control groups (Figure 1). There were no statistically significant differences between the two groups in demographic characteristics (sex, age), disease-related variables (affected side, disease duration, presence of vertigo), vestibular function tests (vHIT, caloric test), or baseline auditory parameters (low-, middle-, high-, and full-frequency hearing thresholds and speech recognition rate) (all *p* > 0.05). These findings confirm the comparability of baseline characteristics, thereby ensuring the validity and reliability of subsequent intergroup comparisons (Table 1).

**Table 1:** Baseline data comparison between the two groups.

Characteristic	Secondary treatment group (*n* = 60)	Control group (*n* = 60)	Statistic	*p*
Gender				
Male	25 (41.67%)	21 (35.00%)	χ^2^ = 0.564	0.453
Female	35 (58.33%)	39 (65.00%)		
Age (years)				
≤50 years	26 (43.33%)	18 (30.00%)	χ^2^ = 0.926	0.336
>50 years	34 (56.67%)	42 (70.00%)		
Affected side				
Right	26 (43.33%)	28 (46.67%)	χ^2^ = 1.690	0.194
Left	34 (56.67%)	32 (53.33%)		
Time (days)				
≤3 days	40 (66.67%)	34 (56.67%)	χ^2^ = 2.936	0.087
>3–7 days	20 (33.33%)	26 (43.33%)		
Vertigo				
Yes	21 (35.00%)	26 (43.33%)	χ^2^ = 0.874	0.350
No	39 (65.00%)	34 (56.67%)		
vHIT				
Normal	44 (73.33%)	35 (58.33%)	χ^2^ = 0.840	0.360
Abnormal	16 (26.67%)	25 (41.67%)		
Caloric test				
Normal	42 (70.00%)	42 (70.00%)	χ^2^ = 1.319	0.251
Abnormal	18 (30.00%)	18 (30.00%)		
Low-frequency (T0) (dBHL, x ± s)	93.46 ± 8.18	92.25 ± 9.18	*t* = 0.210	0.834
Middle-frequency (T0) (dBHL, x ± s)	103.71 ± 10.21	100.91 ± 10.25	*t* = 0.780	0.437
High-frequency (T0) (dBHL, x ± s)	105.96 ± 6.78	102.42 ± 7.76	*t* = 1.543	0.126
All-frequency (T0) (dBHL, x ± s)	101.04 ± 7.42	99.06 ± 7.61	*t* = 1.706	0.091
Speech recognition (T0) Rate (%, x ± s)	0.33 ± 1.11	0.40 ± 1.42	*t* = 0.287	0.775

Caloric Test and vHIT were only performed in patients with vertigo. Inter-group comparisons were conducted using the χ^2^ test (categorical variables) or independent samples t-test (continuous variables). *p* < 0.05 was considered statistically significant. vHIT, Video Head Impulse Test.

### 3.2 Comparison of average hearing thresholds at each frequency and time point between the two groups

#### 3.2.1 Intragroup changes in hearing thresholds in the secondary treatment group

In the secondary treatment group, no statistically significant differences were observed in the low- and high-frequency hearing thresholds between T3 and T1 (all *p* > 0.05); however, the mid-frequency thresholds differed significantly (*p* < 0.05). At T4, the low- and mid-frequency hearing thresholds, but not the high-frequency thresholds, were significantly lower than at T3 (all *p* < 0.05), indicating the onset of marked hearing recovery with more pronounced improvements in the low and mid frequencies. By T5, hearing thresholds across all frequencies, including the high frequencies, had decreased further compared with T4 (all *p* < 0.01), demonstrating that hearing recovery persisted between 8 and 12 weeks after treatment without signs of stagnation. No significant changes in hearing thresholds at any frequency were noted between T6 and T5 (all *p* > 0.05), suggesting that hearing had entered a stable plateau phase.

**Figure 2:**
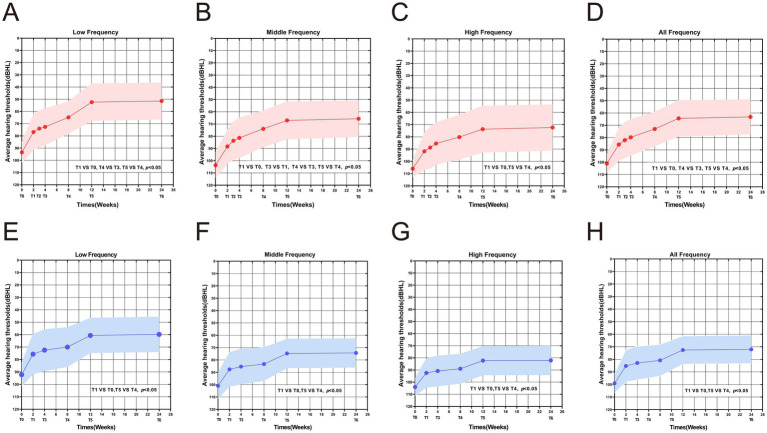
Comparison of average hearing thresholds at different time points. **(A–D)** Serial measurement results of the mean hearing thresholds at low, middle, high, and full-frequency ranges in the secondary treatment group. **(E–H)** Corresponding threshold measurement data of the control group.

Regarding the mean hearing threshold across all frequencies, no significant change was observed at T3 (T3 vs. T1, *p* > 0.05). It began to decline at T4 (T4 vs. T3, *p* < 0.05), decreased further at T5 (T5 vs. T4, *p* < 0.001), and stabilized at T6 (T6 vs. T5, *p* > 0.05) (Figures 2A–D). These findings indicate that secondary treatment induced a sustained and progressive hearing-recovery phase, with the recovery effect stabilizing approximately 12 weeks after treatment.

#### 3.2.2 Intragroup changes in hearing thresholds in the control group

In contrast, the control group showed no significant differences in hearing thresholds between T3 and T1 or between T4 and T3 (all *p* > 0.05), indicating a delayed onset of recovery within the first 8 weeks after initial treatment. At T5, thresholds at all frequencies were significantly lower than those at T4 (all *p* < 0.001), suggesting that recovery began after 8 weeks, later than in the secondary treatment group. No further improvement was observed between T6 and T5 (all *p* > 0.05). Likewise, the mean hearing threshold across all frequencies remained unchanged during T3 and T4 (T3 vs. T1, T4 vs. T3, all *p* > 0.05), decreased significantly at T5 (T5 vs. T4, *p* < 0.001), and stabilized at T6 (*p* > 0.05) (Figures 2E–H). These results collectively indicate that, although both groups eventually reached a plateau of recovery by 12 weeks, the secondary treatment group exhibited an earlier onset and longer effective recovery window.

### 3.3 Comparison of hearing threshold improvement at each frequency and time period between the two groups

**Figure 3:**
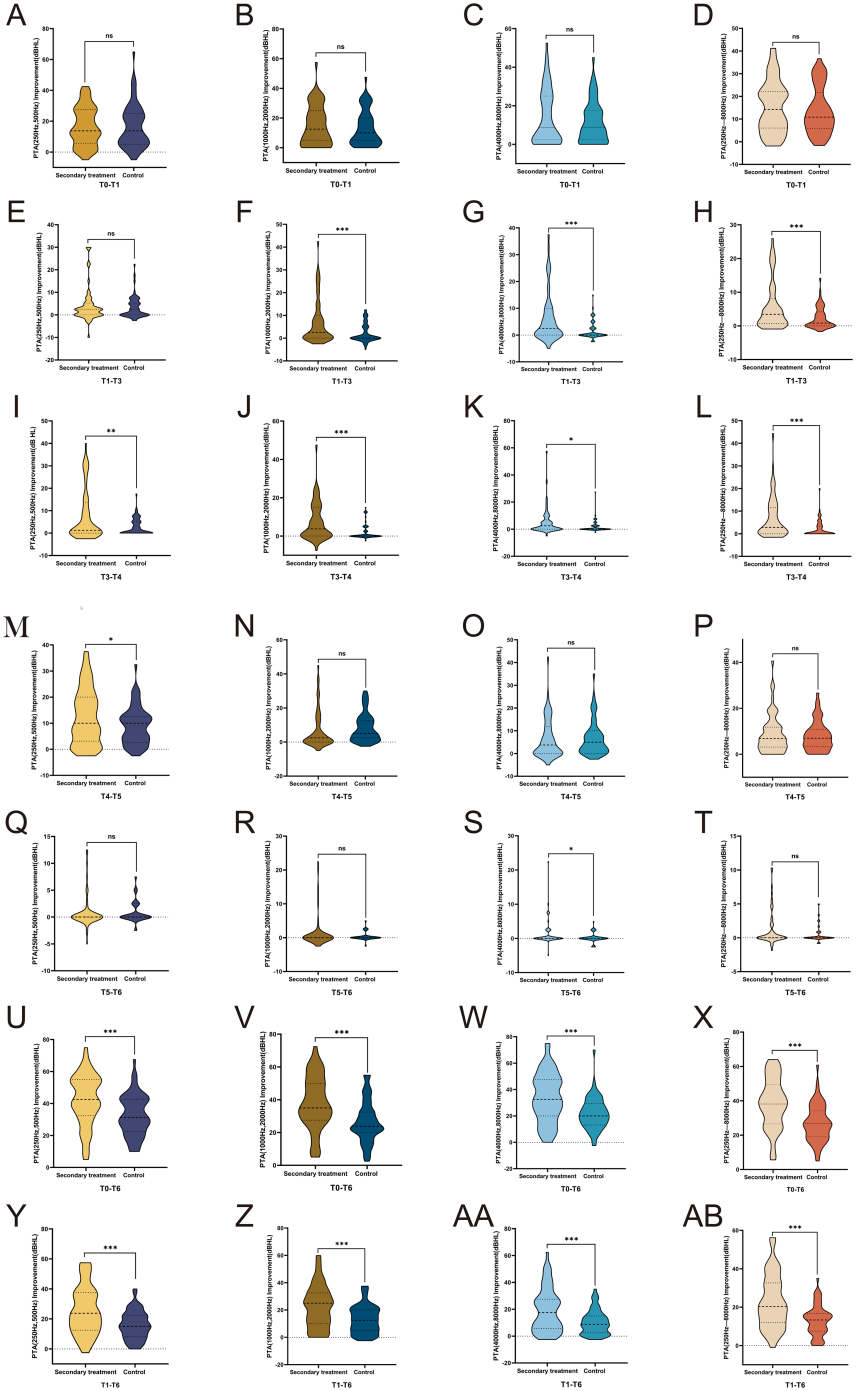
Intergroup differences in hearing threshold improvement across frequencies and time intervals. **(A–D)** Intergroup comparisons of average hearing threshold improvements at low-, mid-, high-, and full-frequency ranges between T1 and T0; **(E–H)** intergroup comparisons of average hearing threshold improvements at each frequency between T3 and T1; **(I–L)** intergroup comparisons of average hearing threshold improvements at each frequency between T4 and T3; **(M–P)** intergroup comparisons of average hearing threshold improvements at each frequency between T5 and T4; **(Q–T)** intergroup comparisons of average hearing threshold improvements at each frequency between T6 and T5; **(U–X)** cumulative comparisons of average hearing threshold improvements at each frequency from T0 to T6; **(Y–AB)** cumulative comparisons of average hearing threshold improvements at each frequency from T1 to T6. *p < 0.05, **p < 0.01, ***p < 0.001, ns, Not significant.

Building on the intragroup analyses, intergroup comparisons revealed distinct recovery patterns. Hearing-threshold improvement was evident in both groups after the first treatment course (T1), with no significant intergroup difference (*p* > 0.05) (Figures 3A–D), thereby maintaining comparability between the groups during the initial phase.

Notably, the secondary treatment group exhibited significantly greater hearing improvement than the control group across most frequencies during the T1–T3 and T3–T4 intervals (all *p* < 0.05) (Figures 3F–L), accompanied by frequency-specific advantages. Specifically, low-frequency hearing recovery did not show significant superiority during the T1–T4 period (*p* > 0.05) (Figure 3E), but an additional significant benefit emerged at T4–T5 (*p* < 0.05) (Figure 3M). In contrast, high-frequency thresholds demonstrated significant advantages at T5–T6 (*p* < 0.05) (Figure 3S).

Furthermore, regarding cumulative hearing recovery from T0 to T6 and from T1 to T6, the secondary treatment group achieved significantly better outcomes across low-, mid-, high-, and overall frequencies (all *p* < 0.001) (Figures 3U–AB), whereas no statistically significant intergroup differences were observed in the other intervals (Figures 3N–R,T).

Collectively, these findings demonstrate that secondary treatment primarily accelerates hearing recovery during the T1–T4 period and enhances long-term cumulative recovery efficacy.

### 3.4 Comparison of recovery rates at different time points between the two groups

**Figure 4:**
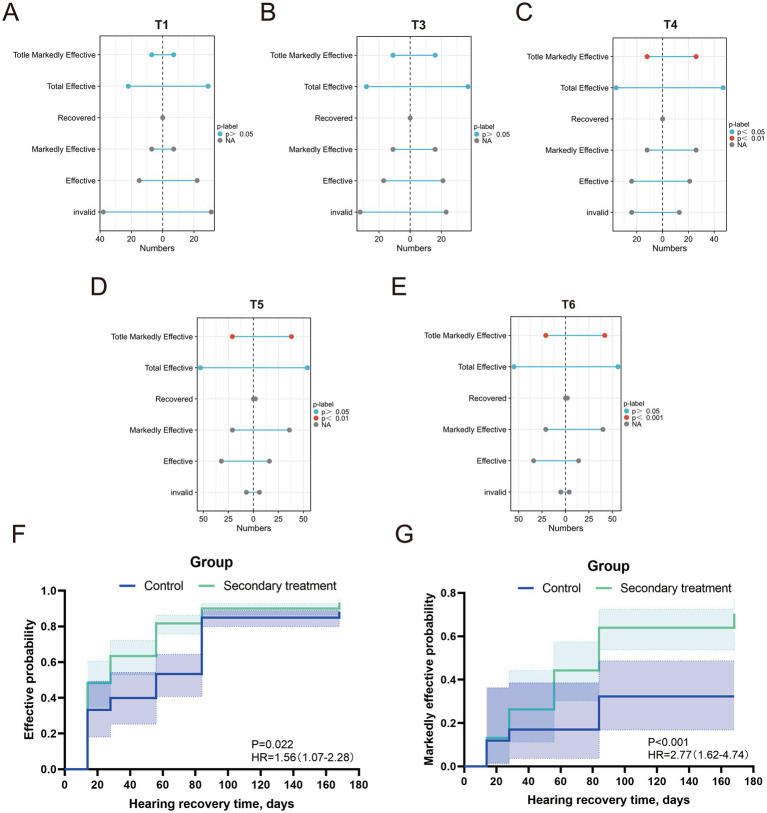
Comparison of hearing recovery effects at different time points and time-to-event outcomes between the two groups. **(A–E)** Distribution characteristics of invalid rate, total effective rate, and marked effective rate at different time points in the secondary treatment group and control group (the right side of the midline represents the secondary treatment group, and the left side represents the control group); **(F–G)** Kaplan–Meier survival curves for achieving ≥15 dB and ≥30 dB improvement in PTA.

Consistent with the threshold analysis, the total effective rate at T4 was significantly higher in the secondary treatment group than in the control group (*p* < 0.05) (Figure 4C). No significant differences were detected at other time points (all *p* > 0.05) (Figures 4A,B,D,E). This pattern indicates that secondary treatment facilitated earlier hearing improvement, whereas the control group showed delayed recovery that became apparent only after T4.

Although the overall effective rate was comparable by the final follow-up, the total marked effective rate remained significantly higher in the secondary treatment group at T4, T5, and T6 (all *p* < 0.01) (Figures 4C–E). These results demonstrate that secondary treatment not only accelerated recovery onset but also enhanced the depth and persistence of hearing improvement. Kaplan–Meier (K–M) survival analysis revealed that the time required for all-frequency PTA to improve by 15 dB was significantly shorter in the secondary treatment group (36.25 days) than in the control group (52.30 days) (*p* = 0.022) (Figure 4F). Furthermore, the time for all-frequency PTA to improve by 30 dB was also shorter in the secondary treatment group (60.67 days), whereas this level of improvement was not achieved in the control group by the end of follow-up (*p* < 0.001) (Figure 4G).

### 3.5 Comparison of PTA-4 at different time points between the two groups

**Figure 5:**
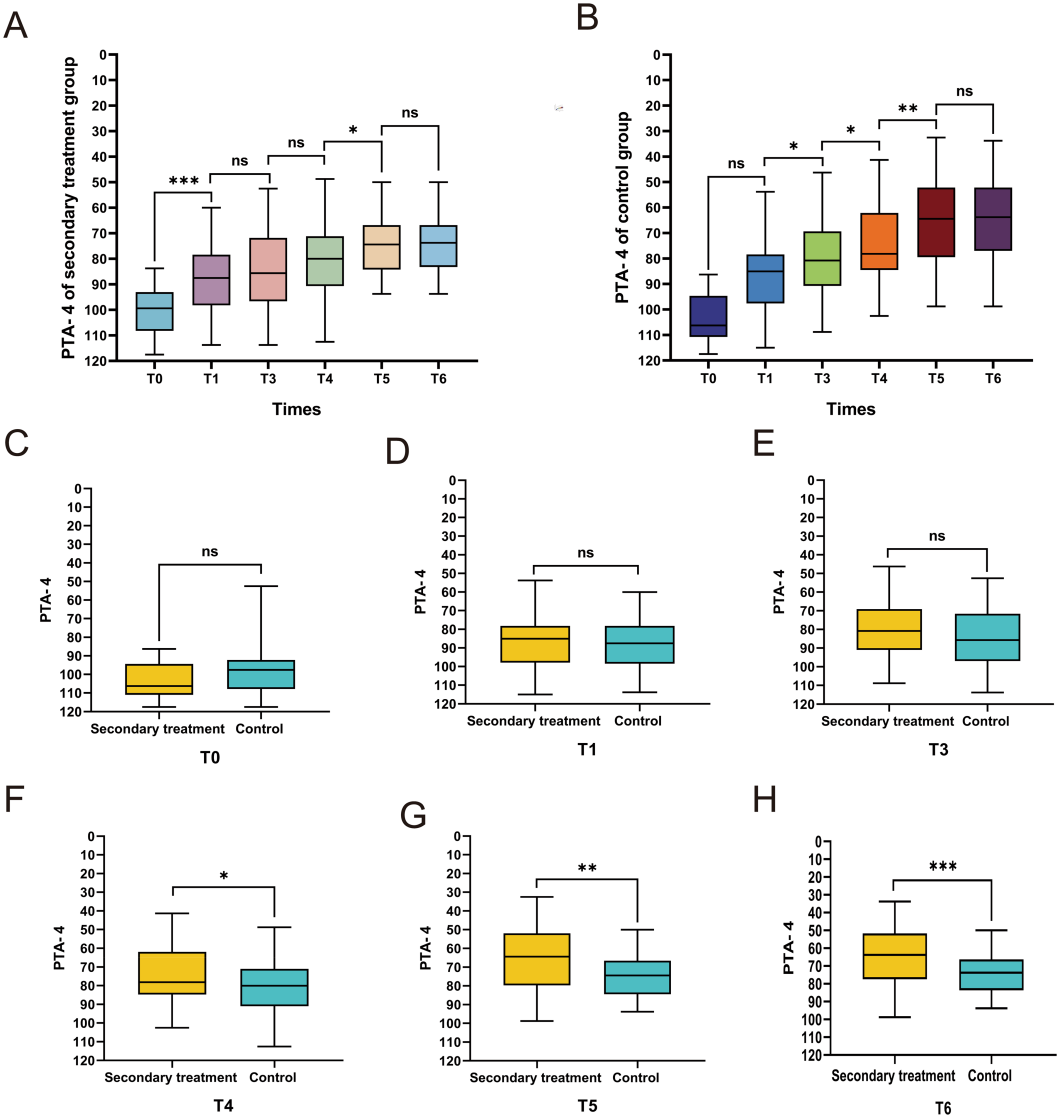
Comparison of pure-tone average of 4 frequencies (PTA-4) between the two groups of patients at different time points. Intragroup trends of PTA-4 changes at different time points in the secondary treatment group **(A)** and control group **(B)**. **(C–H)** Intergroup comparison of PTA-4 at different time points. *p < 0.05, **p < 0.01, ***p < 0.001, ns, not significant.

To further assess functional hearing recovery, we analyzed the PTA-4 of the primary speech-frequency range (500–4,000 Hz). Both groups showed significant improvement at T1 compared with baseline (*p* < 0.05). From T1 to T5, the secondary treatment group demonstrated continuous and significant PTA-4 reductions across successive intervals, whereas the control group exhibited significant improvement primarily between T4 and T5 (all *p* < 0.05). At T6, neither group showed additional improvement relative to T5 (*p* > 0.05) (Figures 5A,B), suggesting stabilization.

Intergroup comparisons revealed no significant differences between the two groups at T0 (*p* > 0.05) (Figure 5C), confirming their baseline comparability. However, from T4 onward, the mean PTA-4 in the secondary treatment group was significantly lower than that in the control group (all *p* < 0.05) (Figures 5F–H), whereas no significant intergroup differences were detected before T4 (all *p* > 0.05) (Figures 5D,E). Collectively, these findings indicate that secondary treatment confers superior hearing recovery within the primary speech-frequency range.

### 3.6 Comparison of speech recognition rate at different time points between the two groups

**Figure 6:**
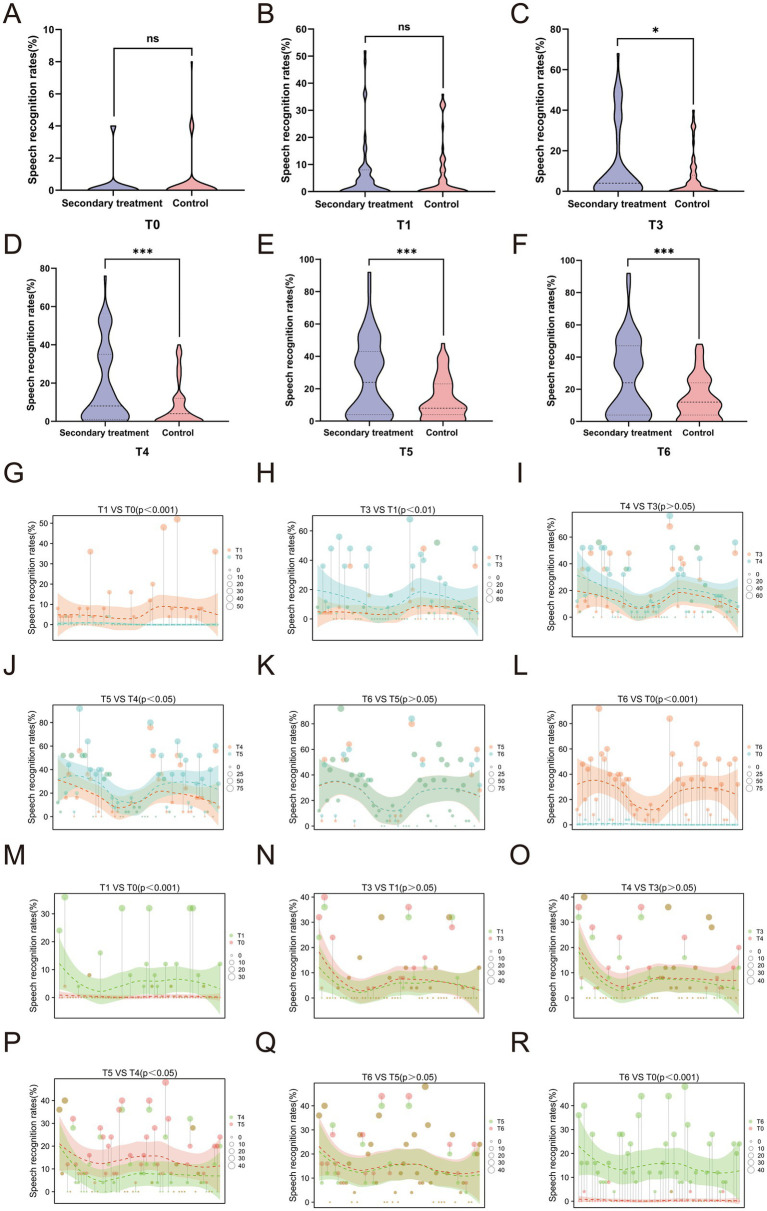
Comparison of speech recognition rates between the two groups of patients at different time points. **(A-F)** Intergroup comparison of speech recognition rate at different time points; **(G-L)** Intragroup trend of speech recognition rate in the secondary treatment group; **(M-R)** Intragroup trend of speech recognition rate in the control group. *p<0.05, ***p<0.001, ns, not significant.

Speech recognition rate, a critical functional indicator of auditory comprehension and communication ability, further illustrated the advantage of secondary treatment. No significant differences were observed between the groups at baseline or T1 (*p* > 0.05) (Figures 6A,B). Beginning at T3, the secondary treatment group demonstrated significantly higher speech recognition rates than the control group, with this advantage persisting through T6 (all *p* < 0.05) (Figures 6C–F). These intergroup differences highlight that secondary treatment not only accelerates hearing recovery but also translates into superior improvement in functional communication outcomes.

Both groups exhibited significant improvements in speech recognition rates at T1 (Figures 6G,M) and T6 (Figures 6L,R) compared with baseline. Although the secondary treatment group did not show a statistically significant change during the T3–T4 interval (*p* > 0.05) (Figure 6I), it demonstrated sustained and progressive gains across the T1–T3 (Figure 6H) and T4–T5 (Figure 6J) periods, reaching a stable plateau after T5 with no further improvement by T6 (Figure 6K). In contrast, the control group showed marked improvement only during the T4–T5 interval (Figures 6N–P), followed by a similar plateau thereafter (Figure 6Q). Collectively, these findings suggest that secondary treatment promotes earlier, more continuous, and more durable recovery of speech comprehension, ultimately resulting in superior long-term auditory and communicative outcomes.

### 3.7 Comparison of THI between the two groups

**Figure 7:**
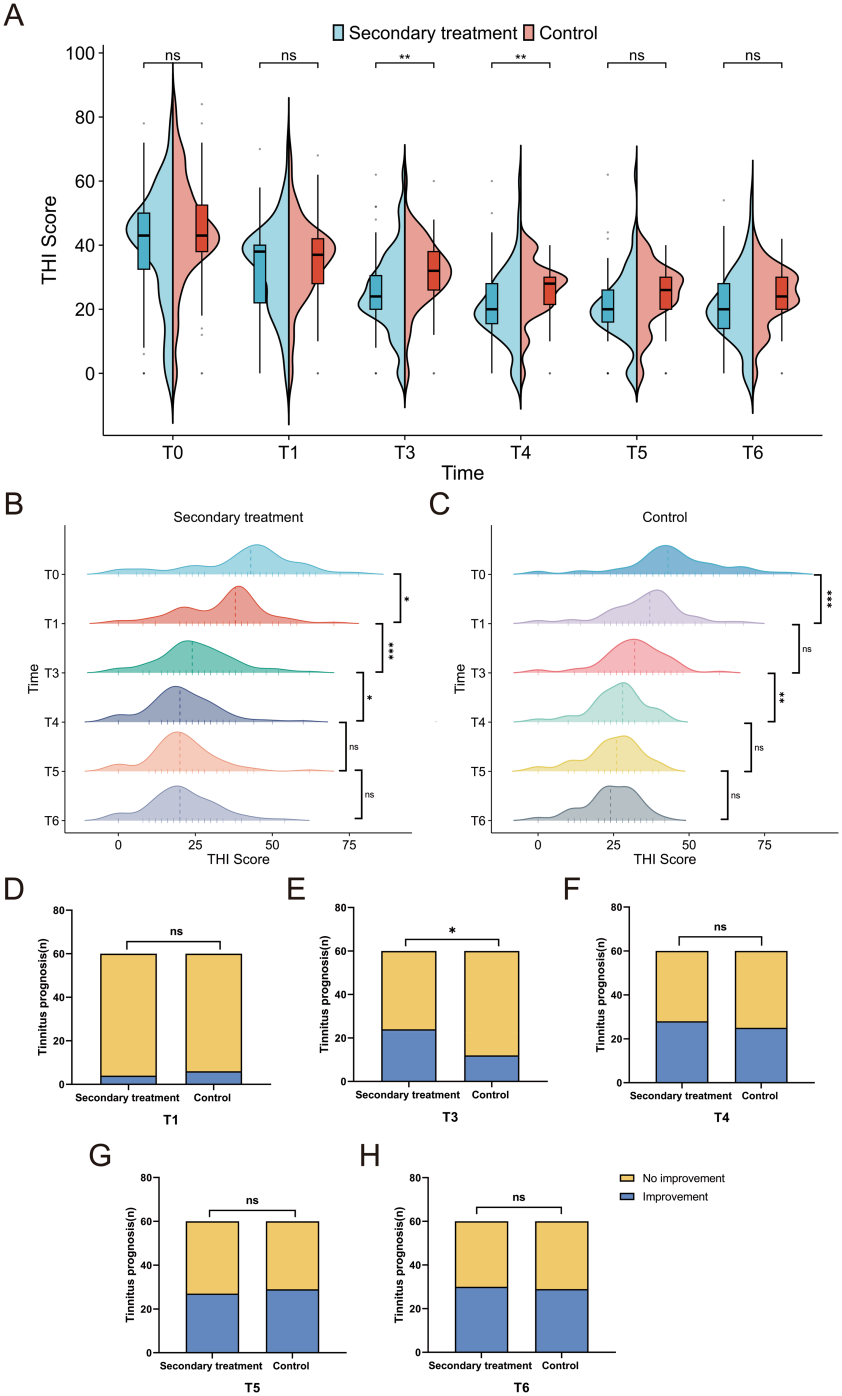
Comparison of tinnitus improvement between the two groups. **(A)** Intergroup comparison of Tinnitus Handicap Inventory (THI) score changes; **(B)** intragroup comparison of THI score changes in the secondary treatment group; **(C)** intragroup comparison of THI score changes in the control group; **(D–H)** comparison of tinnitus improvement rates at different time points. ^*^p < 0.05, ^**^p < 0.01, ^***^p < 0.001, ns: not significant.

Tinnitus efficacy was assessed using the Tinnitus Handicap Inventory (THI) scores and tinnitus improvement rates. The baseline THI scores were comparable between the two groups. At T3 and T4, the secondary treatment group exhibited significantly lower THI scores than the control group (*p* < 0.01), indicating earlier tinnitus relief. From T5 onward, as the control group gradually improved, the intergroup difference narrowed and became statistically non-significant (*p* > 0.05) (Figure 7A). Intragroup analysis revealed that the THI scores in the secondary treatment group decreased steadily from T1 to T4 and stabilized thereafter (Figure 7B), whereas the control group showed improvement only at T1 and T4 (Figure 7C). Regarding the tinnitus improvement rate, the secondary treatment group achieved a significantly higher rate than the control group only at T3 (*p* <0.05) (Figure 7E), with no significant differences at other time points (*p* > 0.05) (Figures 7D,F–H). Collectively, these findings suggest that secondary treatment provides more rapid and substantial tinnitus relief in the short term, whereas the long-term outcomes of the two groups eventually converge.

### 3.8 Univariate analysis of factors influencing hearing recovery following secondary treatment

To further clarify the determinants of treatment efficacy, univariate analyses were conducted. Gender and affected side were not significantly associated with hearing recovery (*p* > 0.05). In contrast, age, disease duration, vertigo, and vestibular function were significant influencing factors. Patients aged ≤50 years, without vertigo, and with normal vHIT and caloric test results demonstrated significantly greater hearing-threshold improvements across all frequencies (all *p* < 0.05). The timing of medical presentation also played an important role. While low-frequency recovery did not significantly differ by presentation time within 7 days (*p* = 0.582), patients presenting within 3 days exhibited greater overall improvement, particularly in the middle- and high-frequency ranges, compared with those presenting between 3 and 7 days. These findings emphasize the importance of early medical intervention for optimal outcomes (Table 2).

**Table 2:** Comparison of hearing threshold improvements across frequencies among different clinical characteristic subgroups in the secondary treatment group at T6 (dBHL, mean ± SD).

Characteristic	*n*	L	M	H	All	L-t (*p*)	M-t(*p*)	H-t (*p*)	All-t (*p*)
Gender									
Male	25	42.80 ± 18.02	41.20 ± 15.48	35.28 ± 17.95	39.76 ± 14.73	0.325 (0.746)	1.279 (0.206)	0.587 (0.560)	0.831 (0.409)
Female	35	41.43 ± 14.61	35.79 ± 16.62	32.43 ± 18.98	36.55 ± 14.77				
Age									
≤50 years	26	51.54 ± 11.40	45.0 ± 14.80	40.67 ± 17.30	45.74 ± 11.49	**4.713 (<0.001)**	**3.106 (0.003)**	**2.727 (0.008)**	**4.062 (<0.001)**
>50 years	34	34.71 ± 15.22	32.72 ± 15.45	28.22 ± 17.69	31.88 ± 14.19				
Affected Side									
Right	26	40.77 ± 16.70	37.98 ± 15.22	31.42 ± 17.27	36.72 ± 13.83	0.287 (0.775)	0.025 (0.98)	0.423 (0.803)	0.532 (0.597)
Left	34	42.94 ± 15.60	38.09 ± 17.22	35.29 ± 19.40	38.77 ± 15.50				
Time									
≤3 days	40	42.81 ± 16.35	41.19 ± 16.02	39.94 ± 16.95	41.31 ± 14.66	0.554 (0.582)	**2.188 (0.033)**	**4.263 (<0.001)**	**2.682 (0.01)**
>3–7 days	20	40.38 ± 15.50	31.75 ± 15.18	20.98 ± 14.68	31.03 ± 12.53				
Vertigo									
Yes	21	31.07 ± 15.56	25.48 ± 14.00	24.40 ± 19.07	26.98 ± 14.60	**4.467 (<0.001)**	**5.315 (<0.001)**	**3.348 (0.001)**	**4.994 (<0.001)**
No	39	47.88 ± 12.95	44.81 ± 13.13	38.58 ± 16.29	43.76 ± 11.08				
vHIT^#^									
Normal	44	46.76 ± 13.61	43.47 ± 13.52	37.32 ± 16.26	42.52 ± 11.68	**4.376 (<0.001)**	**5.125 (<0.001)**	**2.711 (0.009)**	**4.712 (<0.001)**
Abnormal	16	28.91 ± 14.97	23.13 ± 13.80	23.44 ± 20.77	25.26 ± 14.99				
Caloric Test^#^									
Normal	42	46.96 ± 13.51	44.29 ± 13.15	37.73 ± 16.53	42.99 ± 1,159	**4.149 (<0.001)**	**5.594 (<0.001)**	**2.781 (0.007)**	**4.818 (<0.001)**
Abnormal	18	30.42 ± 15.61	23.47 ± 13.34	24.03 ± 19.59	25.97 ± 14.57				

L, Low-frequency; M, Middle-frequency; H, High-frequency; All, All- frequency.

^#^The patients with vertigo underwent caloric test and video head impulse test.Bold values indicate statistically significant differences.

### 3.9 Multivariate logistic regression analysis of factors influencing hearing recovery following secondary treatment

To identify independent predictors of favorable outcomes, multivariate logistic regression analysis was performed. Marked recovery was defined as a ≥30 dB HL improvement in full-frequency hearing threshold (dependent variable = 1), whereas improvement <30 dB HL was classified as general recovery (dependent variable = 0). Independent variables included age, vertigo, caloric test results, and vHIT results—factors identified as significant in univariate analysis. Results revealed that age ≤50 years, absence of vertigo, disease duration ≤3 days, and normal caloric and vHIT results were independent protective factors for achieving marked hearing recovery after secondary treatment (all *p* < 0.05). These findings underscore the prognostic importance of early diagnosis, vestibular integrity, and younger age in optimizing treatment response (Table 3).

**Table 3:** Multivariate logistic regression analysis of influencing factors for delayed hearing recovery (Secondary treatment group).

Independent variable	*β* value	Standard error (SE)	Wald χ^2^ value	*p* value	Odds ratio (OR) value	95% confidence interval (95% CI)
Age (>50 years vs. ≤ 50 years)	0.892	0.326	7.563	**0.006**	2.441	1.283–4.644
Times (3–7 days vs. ≤ 3 days)	0.765	0.351	4.782	**0.029**	2.148	1.068–4.320
Vertigo (Yes vs. No)	1.243	0.385	10.428	**0.001**	3.465	1.682–7.134
Caloric test (Abnormal vs. Normal)	1.057	0.412	6.581	**0.010**	2.878	1.276–6.507
vHIT result (Abnormal vs. Normal)	1.132	0.408	7.765	**0.005**	3.099	1.382–6.938

Statistical significance was defined as *p* < 0.05.Bold values indicate statistically significant differences.

### 3.10 Construction and validation of a prognostic model for patients undergoing secondary treatment

**Figure 8:**
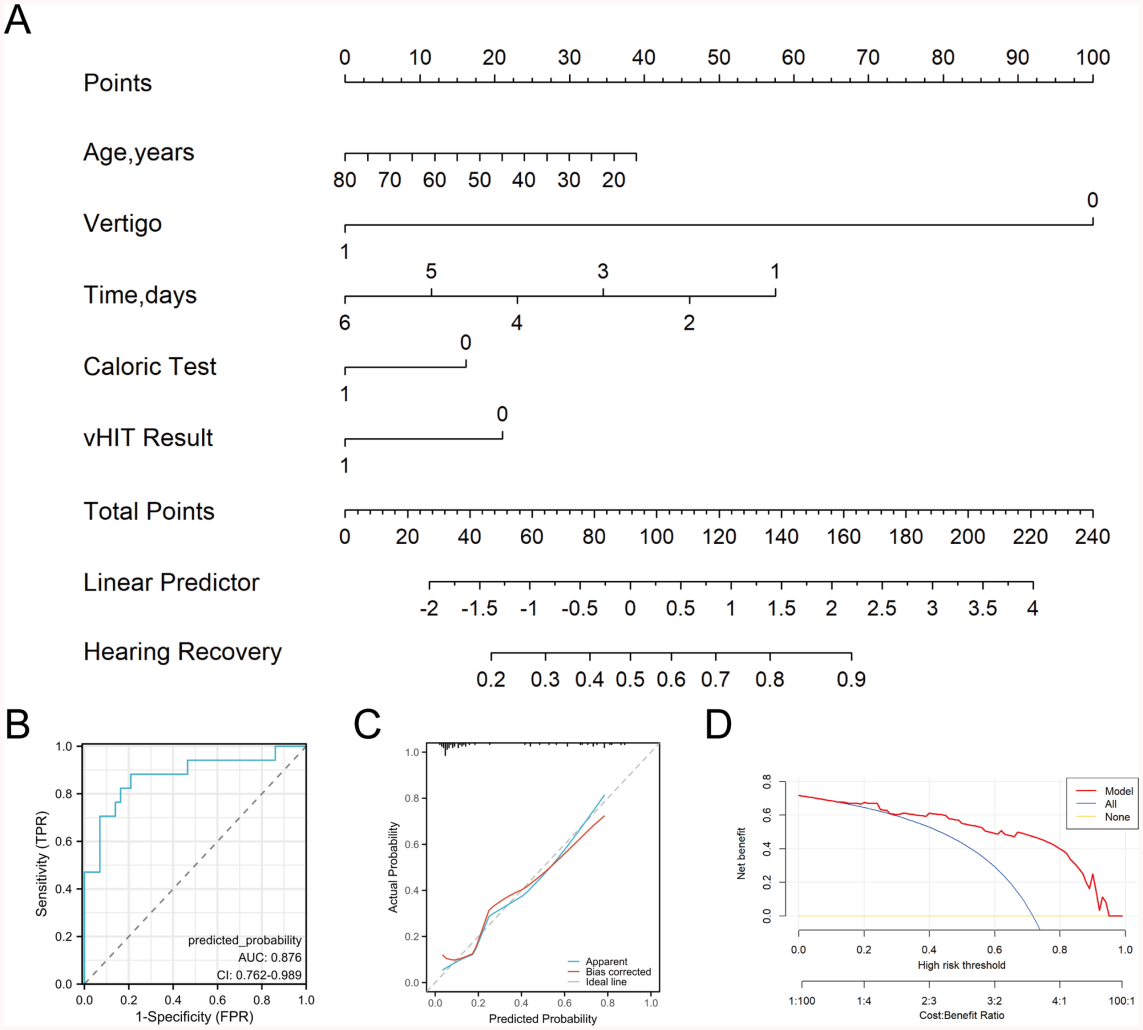
Development and performance assessment of a prognostic nomogram. **(A)** Nomogram of the prognostic prediction model; **(B)** receiver operating characteristic (ROC) curve of the prognostic prediction model; **(C)** calibration curve of the prognostic prediction model; **(D)** clinical decision curve of the prognostic prediction model.

Using the five factors identified by logistic regression as predictors of the efficacy of secondary treatment for total deafness–type SSNHL, we developed a logistic regression model and constructed a nomogram (Figure 8A). ROC curve analysis yielded an area under the curve (AUC) of 0.876 (95% confidence interval [CI]: 0.762–0.989) (Figure 8B). The bootstrap method was applied for internal validation, and the calibration curve demonstrated good agreement with the reference (ideal) line (Figure 8C). The results of the Hosmer-Lemeshow test showed that χ^2^ = 8.621, *p* = 0.375, indicating a good fit of the logistic regression model. DCA indicated that the model provided a positive net benefit across almost the entire range of threshold probabilities, supporting its potential clinical usefulness (Figure 8D).

## 4 Discussion

Total deafness-type SSNHL remains a major clinical challenge due to the profound initial auditory impairment and the generally unsatisfactory response to initial therapy (22–24). Through the follow-up of 120 patients with unilateral total deafness-type SSNHL, this study systematically evaluated the therapeutic efficacy and influencing factors of secondary inpatient treatment. The findings demonstrated that secondary inpatient therapy provided significant clinical benefits for total deafness-type SSNHL and revealed a previously under-recognized “delayed-onset” recovery pattern. Furthermore, the nomogram prediction model developed in this study assigns quantitative scores to the incorporated predictive factors and demonstrates robust predictive performance. By integrating patients’ clinical characteristics with audiological assessment results, it enables an intuitive visualization of individualized prediction outcomes, thereby providing valuable support for clinical decision-making.

The efficacy profile of secondary treatment emphasized the clinical relevance of delayed improvement. Specifically, the therapeutic effects of secondary treatment did not emerge immediately after the completion of the initial course, but became evident at T4, with significant reductions in low- and middle-frequency thresholds compared with T3. Continued improvement was observed through T5, after which hearing stabilized at T6 In contrast, the control group exhibited delayed initiation of hearing recovery, with a shorter “effective recovery window” limited to that single time point. These findings underscore the capacity of secondary treatment to enhance recovery efficiency and provide important evidence for designing long-term clinical follow-up strategies. This “delayed efficacy” characteristic complements previous research findings. Liang et al. demonstrated that early initiation of intratympanic steroid therapy (IST), following failed initial systemic treatment, resulted in significantly improved hearing thresholds at the 6-week follow-up compared to the control group (25). In this study, post-auricular dexamethasone injection was selected over IST primarily due to its minimal invasiveness, favorable safety profile, and avoidance of risks associated with tympanic membrane puncture (e.g., infection, perforation). Additionally, this administration route is compatible with the combined salvage treatment regimen of the present study, and previous studies have validated that post-auricular glucocorticoid injection can improve the therapeutic efficacy of SSNHL and alleviate associated symptoms (26). Similarly, Lee et al. observed, in a retrospective analysis of 110 patients, that hyperbaric oxygen salvage therapy achieved an efficacy rate of 38.9% at 3-month assessment (27). However, existing studies have either overlooked the time-lagged nature of therapeutic benefits or employed singular salvage regimens without comprehensive treatment approaches. By monitoring seven consecutive time points, the present study was the first to precisely delineate that the “onset window of therapeutic efficacy” for secondary treatment occurs between 4 and 12 weeks following the first hospitalization. This conclusion aligns with the findings of Lee et al., who observed that SSNHL patients may achieve delayed recovery within 3 months (27), thereby confirming that secondary treatment can advance the initiation of recovery by approximately 4 weeks compared with the control group. Furthermore, Kim et al. reported a case in which partial hearing improvement was achieved approximately 300 days after onset following secondary treatments including acupuncture and IST (28). These findings provide critical evidence against premature conclusion of treatment futility in clinical practice.

From the perspective of frequency specificity, the magnitude of hearing improvement achieved by secondary treatment was significantly greater at low and middle frequencies than at high frequencies (at T6: low-frequency, 42.00 ± 15.98 dB HL; middle-frequency, 37.50 ± 16.25 dB HL; high-frequency, 33.62 ± 18.46 dB HL). This frequency-dependent difference is closely associated with cochlear anatomy and physiology. Hair cells mediating low- and middle-frequency perception are located in the apical turn of the cochlea, an area supported by extensive collateral circulation. These regions are particularly responsive to comprehensive therapeutic regimens such as hyperbaric oxygen (enhancing inner ear oxygenation), post-auricular corticosteroid injection, and microcirculatory agents. Conversely, high-frequency hair cells are located in the basal turn and primarily rely on the labyrinthine artery for blood supply. In total deafness-type SSNHL, thrombosis or embolic occlusion of this artery often results in irreversible cochlear damage (29). This finding contrasts with the conclusion of Sun et al., who proposed that IST is more beneficial for high-frequency recovery (30). The discrepancy likely stems from differences in patient selection, as Sun et al. investigated high-frequency descending-type SSNHL, rather than the total deafness-type analyzed in the present study. These observations suggest that distinct SSNHL subtypes differ substantially in their pathophysiological mechanisms and therapeutic responsiveness, highlighting the necessity of precision-tailored interventions to promote high-frequency recovery in total deafness-type SSNHL.

Importantly, the clinical value of secondary treatment is reflected not only in the magnitude of hearing threshold improvement but also in the depth of functional recovery. Although no statistically significant difference was observed in total effective rate between the two groups at T6, the total marked effective rate in the secondary treatment group was significantly higher than in the control group beginning at T4 (T4: 43.3% vs. 20.0%, *p* = 0.006; T5: 63.3% vs. 35.0%, *p* = 0.002; T6: 70.0% vs. 35.0%, *p* < 0.05). Furthermore, the mean speech-frequency threshold at T6 was markedly lower in the secondary treatment group (65.31 ± 14.32 dB HL vs. 73.88 ± 11.18 dB HL, *p* < 0.05). Beyond threshold recovery, secondary treatment also enhanced the auditory processing capacity of the central auditory pathway, resulting in significantly greater improvement in speech recognition (T6: 26.53 ± 23.20% vs. 14.27 ± 13.02%, *p* < 0.05). These findings underscore the therapeutic potential of secondary treatment to restore not only hearing sensitivity but also speech perception, thereby addressing the persistent clinical dilemma of patients who can hear but cannot understand. Emerging evidence similarly indicates that the primary objective of SSNHL management should extend beyond audiometric thresholds to include meaningful improvement in speech comprehension, which is consistent with the findings of this study (31, 32).

Regarding tinnitus, secondary treatment yielded more pronounced early relief of tinnitus symptoms (T3) compared with the control group (T3 THI score: 25.47 ± 12.36 vs. 31.47 ± 10.58, *p* = 0.005; tinnitus improvement rate: 40.00% vs. 20.00%, *p* = 0.017). Although the THI scores of the secondary treatment group remained numerically lower beyond T5, the between-group difference was no longer statistically significant (*p* > 0.05). This pattern partially accords with the findings of Peng and Diao et al., who proposed that tinnitus improvement following SSNHL is time-dependent and may gradually occur in the control group through endogenous compensatory mechanisms during long-term follow-up (33, 34). Nevertheless, the early alleviation of tinnitus achieved by secondary treatment has substantial clinical relevance. Early symptom relief can mitigate psychological distress such as anxiety and insomnia, creating a favorable physiological and psychological milieu that supports subsequent auditory recovery. This represents an important contribution of secondary treatment to holistic auditory rehabilitation.

One of the major clinical challenges in the secondary treatment of total deafness-type SSNHL lies in the absence of reliable predictors of treatment efficacy, which often results in either overtreatment or missed therapeutic opportunities. Through univariate and multivariate logistic regression analyses, this study identified age, disease duration, vertigo, and vestibular function as independent factors influencing the efficacy of secondary treatment. These findings provide a quantitative basis for identifying patient populations most likely to benefit from secondary therapy. Age ≤50 years was found to be a significant protective factor for treatment efficacy (OR = 0.409, 95% CI: 0.158–0.673, *p* = 0.006). Patients in this subgroup demonstrated significantly greater full-frequency hearing improvement at T6 (45.74 ± 11.49 dB HL) compared with those aged >50 years (31.88 ± 14.19 dB HL, *p* < 0.001). This observation is consistent with the findings of You et al. in 75 pediatric SSNHL cases, where younger patients exhibited greater regenerative capacity of cochlear hair cells and neural tissues, rendering them more responsive to the anti-inflammatory and reparative effects of corticosteroids. Conversely, older patients tend to show diminished responsiveness due to age-related degenerative changes in the auditory pathway, including hair cell loss and myelin sheath damage (35). Similarly, Yang et al. reported a 3.07-fold higher efficacy rate in SSNHL patients aged ≤50 years compared to older individuals (36), underscoring age as a critical determinant of outcomes in the secondary treatment of total deafness-type SSNHL. These findings underscore the need for clinicians to establish realistic pretreatment expectations for patients over 50 years and prioritize post-intervention auditory rehabilitation, such as speech perception training and auditory integration exercises, to optimize functional outcomes.

Patients with a disease duration ≤3 days exhibited significantly better outcomes with secondary treatment (OR = 0.466, 95% CI: 0.136–0.615, *p* = 0.029). The influence of disease duration also varied across frequencies: low-frequency recovery was not significantly constrained by disease duration within 7 days, whereas improvements in mid- and high-frequency hearing were markedly greater in patients with a duration ≤3 days. This result refines the conventional clinical principle of “early intervention.” While prior studies generally indicated that a disease duration ≤14 days predicts better prognosis (37, 38), the present findings further demonstrate that in total deafness-type SSNHL, mid- and high-frequency hearing are particularly time-sensitive. Initiating secondary treatment within 3 days of onset can substantially reduce the risk of irreversible high-frequency damage. The present study, however, refines this temporal threshold to 3 days, thereby offering a more precise clinical reference for determining optimal treatment initiation timing.

Vertigo and abnormal vestibular function were identified as significant negative predictors of secondary treatment efficacy. Patients presenting with vertigo demonstrated markedly lower full-frequency hearing improvement (26.98 ± 14.60 dB HL) than those without vertigo (43.76 ± 11.08 dB HL, *p* < 0.001). Similarly, abnormal vHIT results (OR = 3.099, 95% CI: 1.382–6.938, *p* = 0.005) and caloric test abnormalities (OR = 2.878, 95% CI: 1.276–6.507, *p* = 0.010) were associated with poorer hearing recovery across all frequencies. This finding aligns with previous studies indicating that the presence of vertigo is associated with a less favorable prognosis (39–41). In clinical settings, the extent of inner ear injury can be rapidly assessed via vHIT and caloric testing. For patients exhibiting abnormal vestibular responses, the potential benefit of secondary treatment should be carefully weighed, and adjunctive therapies such as vestibular rehabilitation or extended treatment duration may be considered to improve recovery outcomes.

Although this study validated the therapeutic efficacy of secondary intervention, identified key prognostic factors, and developed a prognostic prediction model with favorable predictive performance, several methodological limitations should be acknowledged. First, the single-center and non-randomized design inherently introduces the risk of selection bias, thereby limiting the external validity and generalizability of the findings. Second, the absence of a placebo-controlled group warrants attention. Considering the potential for spontaneous recovery in idiopathic SSNHL, this limitation may confound the interpretation of treatment-related effects, and the potential influence of placebo effects on the observed auditory recovery cannot be entirely excluded. Collectively, these limitations underscore the need for future multicenter, randomized, placebo-controlled trials to more rigorously delineate the true therapeutic efficacy of secondary intervention.

## 5 Conclusion

Despite these constraints, the findings of this study provide robust evidence that secondary inpatient treatment for total deafness-type SSNHL accelerates the onset of hearing recovery and enhances its overall extent, particularly within low- and mid-frequency ranges and in speech recognition performance. Clinically, secondary treatment should be prioritized for patients aged ≤50 years, with a disease duration ≤3 days, absence of vertigo, and normal vestibular function as evidenced by vHIT and caloric tests, as these individuals exhibit the greatest likelihood of favorable outcomes. Continuous follow-up until at least 12 weeks post-treatment (T5 phase) is recommended to avoid premature cessation of therapy based on transiently limited improvement observed at earlier evaluations. For patients older than 50 years or those presenting with concomitant vestibular dysfunction, adjunctive therapies—including neurotrophic agents and vestibular rehabilitation—may be considered to optimize auditory outcomes. Collectively, these findings, together with the developed visual nomogram prediction model, provide an evidence-based framework for guiding clinical decision-making regarding the secondary treatment of total-deafness–type SSNHL, thereby promoting the formulation of more precise therapeutic strategies and the improvement of patient outcomes.

## Data Availability

The raw data supporting the conclusions of this article will be made available by the authors, without undue reservation.
